# Effect of *Tricholoma matsutake* Powder and Colored Rice Flour on Baking Quality and Volatile Aroma Compound of Cookie

**DOI:** 10.3390/foods14132182

**Published:** 2025-06-22

**Authors:** Yuyue Qin, Shu Wang, Haiyan Chen, Yongliang Zhuang, Qiuming Liu, Shanshan Xiao, Charles Brennan

**Affiliations:** 1 Yunnan International Joint Laboratory of Green Food Processing, Faculty of Food Science and Engineering, Kunming University of Science and Technology, Kunming 650550, China; rabbqy@163.com (Y.Q.); wangshu062211@163.com (S.W.); seacome@163.com (H.C.); kmylzhuang@163.com (Y.Z.); kgqml2012@163.com (Q.L.); 2 School of Science, Royal Melbourne Institute of Technology, Melbourne 3000, Australia

**Keywords:** cookie, *Tricholoma matsutake*, colored rice, baking quality, aroma

## Abstract

In recent years, the consumers’ demand for healthy foods has been increased. To address the dietary related diseases, the food products enriched with mushroom or colored rice were promoted. The effects of *Tricholoma matsutake* powder and colored rice flour on baking quality and volatile aroma compound of cookies were investigated. Texture analyzer, and electronic nose (e-nose) were used to analyze the physicochemical, structural, and digestibility properties and volatile aroma compound of cookie. With the content of *Tricholoma matsutake* powder and colored rice flour increased, the hardness and free amino acid content increased. Cookie in terms of weaker network structure, relatively crispy cookie texture, and better in vitro digestion activity was obtained with appropriate amount replacement. The cookie sample contained with 5% *Tricholoma matsutake* and 20% red rice exhibited acceptable hardness and lowest starch hydrolysis rate. The volatile aroma compounds were also affected by the wheat flour substitution. The results indicated that *Tricholoma matsutake* powder and colored rice flour substitution improved the baking quality of cookie.

## 1. Introduction

Cookie is widely consumed around the world due to its convenience, long shelf life, low cost, and ease of use. It is also considered as the right product for food enrichment in most of the developed countries [1]. The formulation of cookie mainly contains wheat flour, fat, and sugar. To address the dietary related diseases, the food products enriched with various bioactive components like mushroom and colored rice were promoted. Composite flour with bioactive components could be used for cookie to add its nutritional and functional value, due to the consumers’ demand for healthy food products. Mushroom is also abundant of gluten-free and high-quality protein. Recently, mushroom powder like *Dictyophora indusiata*, *Pleurotus eryngii*, and *Cantharellus cibarius* was added to noodle, pizza crust, and bread as a natural ingredient to develop nutritious and hypoglycaemic functional food product [2,3].

*Tricholoma matsutake* is a valuable edible mushroom and mainly grows in Asia, North America, and Europe. Yunnan Province, China is one of the major production areas, and it accounts for a substantial portion of the national export of *Tricholoma matsutake* mushrooms. In 2022, Yunnan Province exported 174,967 t of matsutake mushrooms [4]. *Tricholoma matsutake* is an excellent source of nutrient and contains polypeptide, polysaccharide, and matsutakeol, all of which have biological activity, such as antioxidant, blood fat-reducing, anti-tumor, and immunoregulatory activity [5]. So, it is worthy of further study for preparing functional foods. It is welcome by consumers for its particular spicy-aromatic aroma [6]. *Tricholoma matsutake* mushroom could provide a better resource of gluten-free and high-quality protein for the bakery industry. Furthermore, cookie enriched with dietary fiber attracted great interest by both producers and researchers. In recent years, since *Tricholoma matsutake* mushroom was a valuable nutrient for human health, *Tricholoma matsutake*-based foods were sold in the market. In our previous study, *Tricholoma matsutake* was used as raw material to prepare bread (data not published). From the view of taste, texture, and aroma, 5% *Tricholoma matsutake* replacement was preferred. So, 5% *Tricholoma matsutake* was selected in this study.

Colored rice including black rice, purple rice, and red rice is widely consumed in China, especially in Yunnan Province. Colored rice is a good source of phenolic compound and anthocyanin, and could be added to substitute part of wheat flour for bakery food production [7]. It is also abundant in dietary fiber, contributing to effective reduction in cholesterol level of human body. Bread fortified with anthocyanin-rich extract from black rice was demonstrated to be a kind of low GI food, because that its anthocyanin could inhibit carbohydrate-digesting enzymes, including α-glucosidase and α-amylase [8]. It has been reported that a moderate content of roselle powder and purple rice flour replacement can improve bread quality, especially positively affecting its flavor [9]. We prepared bread, noodle, and flower cake with mushroom powder and colored rice flour, the upper limit of replacement was suggested to be 25% [1,2,3,9].

Published data on the combination addition of *Tricholoma matsutake* powder and colored rice flour in cookie processing is still limited. Previous studies have focused primarily on the individual health benefits of *Tricholoma matsutake* and colored rice, but there is a significant gap in research regarding their combined application in food products, especially in cookie formulations. This gap is particularly important because, despite their potential health benefits as natural food ingredients, both *Tricholoma matsutake* and colored rice face significant challenges in practical applications. The color, crispness, taste, and aroma value of cookie might be partially decreased with the addition of raw materials with higher dietary fiber content. In this study, the *Tricholoma matsutake* powder and colored rice flour (10, 15, and 20% black, purple, or red rice) were added to cookie formulation, and physiochemical properties and sensory attributes of cookies were also determined.

## 2. Materials and Methods

### 2.1. Materials and Chemical Reagents

*Tricholoma matsutake* and black, purple, and red rice were purchased from Kunming local market, China. The dried *Tricholoma matsutake* mushroom and colored rice were milled and then sieved through a 60 mesh screen to obtain *Tricholoma matsutake* powder and colored rice flour. The milling process was conducted using a Yun Bang model YB-1000A multifunctional grinder, manufactured by Yongkang Shufeng Industry and Trade Co., Ltd., Yongkang, Zhejiang, China. This device operates at 220 V, 3100 W power, and a motor speed of 35,000 RPM, which was suitable for achieving the desired particle size of 60–350 mesh. Low-gluten wheat flour (Meimei brand, 8 g/100 g protein; 72 g/100 g carbohydrate) and high-gluten wheat flour (Golden Statue brand, 13.5 g/100 g protein; 72 g/100 g carbohydrate) were purchased from Nanshun Food Co., Ltd., located in Changzhou, China. The protein and carbohydrate contents of the flours were provided by the manufacturer. Butter, milk powder, sugar, salt, and egg were purchased from a local supermarket in Kunming, China. Other chemical reagents were obtained from Sigma-Aldrich Co., Ltd. (St. Louis, MO, USA) and were of analytical grade.

### 2.2. Cookies Preparation

#### 2.2.1. The Recipe of Cookies

The recipe of cookies was listed in Table 1. Instead of the total amount of high-gluten flour (130 g), 5% *Tricholoma matsutake* powder (T5) and 10, 15, and 20% black rice flour (T5B10, T5B15, and T5B20 sample), 5% *Tricholoma matsutake* powder (T5) and 10, 15, and 20% purple rice flour (T5P10, T5P15, and T5P20 sample), and 5% *Tricholoma matsutake* powder (T5) and 10, 15, and 20% red rice flour (T5R10, T5R15, and T5R20) were substituted.

#### 2.2.2. Cookies Production

The cookie was prepared as follow: Firstly, the butter was softened. Secondly, sugar was mixed with butter in a SM-201 Mixer (SINMAG Machinery Co., Ltd., Shanghai, China) and stirred until pale color. Then, *Tricholoma matsutake* powder, black rice flour, and other ingredients were put into the mixer and mixed at medium speed until no dry powder. The cookie dough was stored at −20 °C for 60 min. After the cookie dough turned firmer, it was taken out and cut into pieces in size of 20 mm × 20 mm × 8 mm. The cookie dough was heated in an oven (C40 Oven, Hauswirt Baking Appliance Co., Ltd., Shenzhen, China) at 175 °C for 15 min until the cookie surface turned to golden brown. Finally, the cookies were taken out, cooled to room temperature, sealed with package, and stored at room temperature avoid light.

### 2.3. Physical Properties of Cookies

#### 2.3.1. Colorimetric Analysis 

The surface color of the cookies sample was determined using a colorimeter (WSC-S, Precision Science Co., Ltd., Shanghai, China). The color parameters L* (brightness), a* (red-green), and b* (yellow-blue) were recorded. These parameters are part of the CIELAB color space, where L* represents the lightness of the color, a* represents the red-green axis, and b* represents the yellow-blue axis. The test was repeated in triplicate.

#### 2.3.2. Texture Analysis

The hardness of the cookies was determined by a texture analyzer (TA-XT Plus, SMS, Godalming, Surrey, UK) [9]. The settings were: P/2 for the probe, 5 g of trigger force, and 10 mm of compression distance. The speed was 2 mm/s pre-test, 1 mm/s during the test, and 2 mm/s post-test. Data acquisition and analysis were performed using Exponent software, version 6.1.23.0 (Stable Micro Systems Ltd., Godalming, Surrey, UK). Each sample was tested no less than six times.

### 2.4. In Vitro Digestion Analysis

The in vitro digestibility of the cookies was analyzed using the method described in [3]. Initially, 0.5 g of each cookie sample was weighed and mixed with 3 mL of pepsin solution (7 mg/mL, prepared in 0.5 mol/L HCl-KCl buffer, pH = 1.5). This mixture was incubated at 37 °C in a water bath for 30 min to simulate the gastric phase. Subsequently, 20 mL of sodium acetate-acetic acid buffer solution (0.5 mol/L, pH = 5.2) was added, and the mixture was further incubated at 37 °C for an additional 5 min. Then, 2 mL of pancreatic α-amylase (370 U/mL, prepared in 0.5 mol/L sodium acetate-acetic acid buffer, pH = 5.2) and 0.1 mL of amyloglucosidase solution (500 U/mL) were introduced. The mixture was incubated at 37 °C, and at digestion times of 0, 20, 60, 90, 120, 150, and 180 min, 1 mL of the enzymatic solution was sampled. To terminate the reaction, 4 mL of anhydrous ethanol was added to each sample, which was then centrifuged at 4000 rpm for 10 min. The supernatant was collected, and the glucose content in the samples was determined using the GOPOD method. The in vitro digestibility of the cookies was expressed as %. The test was repeated in triplicate and the data were expressed as mean value ± standard deviation (SD).

### 2.5. Determination of Free Amino Acids

The free amino acid content of the cookies was determined using a specific method [10]. About 0.1 g of cookies was weighed and mixed with 25 mL of 50 g/L trichloroacetic acid. After mixing, the solution was left to stand for 1 h and then centrifuged at 10,000× *g* for 15 min. The supernatant was collected and filtered through a 0.22 μm filter membrane. Amino acid analysis was subsequently performed using an amino acid analyzer (L-8900, Hitachi, Tokyo, Japan). The test was repeated in triplicate.

### 2.6. Aroma Analysis

The aroma of cookies was analyzed using the E-nose analysis system (Bosin Industrial Development Co., Ltd., Shanghai, China). The system contains 18 metal oxide sensors with different sensitivities to the measured gases. Approximately 4 g of smashed cookies was placed into a 40 mL flask and left for 30 min at 38 °C. The E-nose was set with the following parameters: Humidity: 55%, temperature: 25 °C, inlet preparation time: 5 s, flow rate: 1000 mL/min, and determination time: 60 s [10]. To ensure the reliability of the results, each sample was analyzed in triplicate, meaning the measurements were repeated three times for each sample. The average values from these replicates were used for further analysis. The cookies’ aroma was also analyzed by the Principal Component Analysis (PCA) method.

### 2.7. Sensory Evaluation

The sensory profiling was followed by ISO 13299:2016 [11] and China National Standard GB/T 12311–2012 for sensory analysis methodology [3]. The evaluation was conducted in a controlled food sensory laboratory by a trained panel of 16 assessors (including8 males and 8 females, aged 18–50) from the Faculty of Food Science and Engineering, Kunming University of Science and Technology. All the panelists had prior sensory evaluation experience and received a 20 h standardized assessor training program, which contained basic taste recognition, texture assessment, and protocol familiarization. Informed consent was obtained from all volunteers. The sensory evaluation members were asked to rate their preference of how they like the color, appearance, odor, taste, and texture of the cookies by indicating a numerable value to rate their preference from one expressing extremely bad to nine excellent. They were also asked to rate their preference of how much they would like to purchase the products via nine points overall acceptability from one (dislike extremely) to nine (like extremely). The test was repeated in triplicate.

### 2.8. Statistical Analysis

SPSS 21.0 software was used to calculate means and standard deviations for statistical analysis. Duncan’s test was performed for One-way ANOVA (*p* < 0.05). The Pearson correlation among hardness, color, sensory score, and starch hydrolysis rate was analyzed by Origin 2021 software. Each experiment was repeated three times. The significance level for all tests was set at α = 0.05. Any *p*-values less than 0.05 were considered statistically significant.

## 3. Results

### 3.1. Color

**Figure 1:**
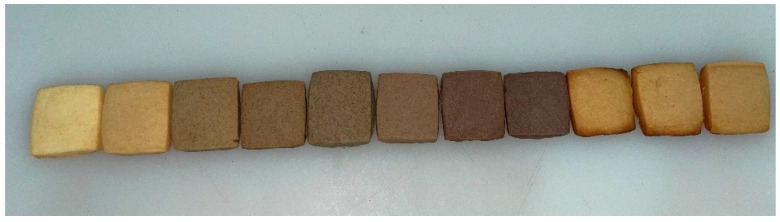
Different Recipes of Cookies (Labeled from Left to Right as CON, T5, T5B10, T5B15, T5B20, T5P10, T5P15, T5P20, T5R10, T5R15, T5R20).

The color of the bakery product is influenced by some aspects, including baking time, baking temperature, and the recipe of cookies. Color has a big impact on consumer choice. The color changes for cookies with *Tricholoma matsutake* powder and colored rice flour substitution were shown in Figure 1 and Table 2. *Tricholoma matsutake* powder with brown color and colored rice flour with black, purple, or red color were darker than wheat flour with white color. As for L* value, cookies with colored rice were significant (*p* < 0.05) lower than that without colored rice substitution (T5 sample). For the same amount of substitution, such as T5B15 sample with black rice flour, T5P15 sample with purple rice flour, and T5R15 sample with red rice flour, there was no significant (*p* > 0.05) difference between T5B15 and T5P15 sample. This might be due to the similar dark color for black rice and purple rice. However, the L* value of T5B15 and T5P15 sample was significant (*p* < 0.05) lower than that of T5R15 sample, due to the fact that red rice flour with light red color. There was no significant (*p* > 0.05) difference among cookie samples with 10%, 15%, and 20% red rice flour substitution.

As for a* value, T5 sample (7.01 ± 0.37) with *Tricholoma matsutake* powder was significant (*p* < 0.05) higher than the control sample (4.75 ± 0.11), owing to the red-green color from *Tricholoma matsutake* powder. The a* value of cookies with black rice and purple rice was significant (*p* < 0.05) lower than that of T5 sample. However, the red rice flour substitution did not affect a* value of cookies. There was no significant (*p* > 0.05) difference among cookie samples with 10%, 15%, and 20% all colored rice flour substitution. This was due to the difference in red-green color for black, purple, or red rice flour. A similar trend was also found in b* value (yellow-blue).

Furthermore, the caramelization and Melad reaction occurred during the baking process also would result in deeper color of cookies [9]. To achieve a popular color for consumers, less baking time was preferred for cookies with the *Tricholoma matsutake* mushroom substitution or colored rice substitution. So, the color of all samples was acceptable to the evaluation panel during the sensory evaluation. Whatever the *Tricholoma matsutake* mushroom substitution or colored rice substitution, the color of cookies was attractive color for consumers.

### 3.2. Texture

For cookie acceptance, proper crispness is very important to the consumers, owing to the fact that a certain descent in hardness improves the acceptability of cookies through providing a better taste. The evaluation of TPA on the hardness was listed in Table 2. The order of cookie hardness was as follows: CON > T5B20, T5 > T5P20 > other cookie samples. The hardness of control sample (1602 ± 147 g) without substitution was the highest. With the replacement of 5% *Tricholoma matsutake* powder, the hardness of the cookies was significantly (*p* > 0.05) lowered (T5 sample: 1194 ± 74 g). *Tricholoma matsutake* mushroom was abundant of dietary fiber. The mushroom powder substitution of wheat flour reduced the content of gluten and inhibited the formation of the gluten network, resulting in a reduction in the strength of the gluten protein network [12]. It was reported that an increase in betel leaves powder led to an increase in cookie dough hardness, which was attributed to the increased protein content and its interaction during dough preparation and cookie baking [13].

The hardness of cookies with colored rice replacement was also significantly (*p* > 0.05) lowered. This might be due to that the gluten protein content of colored rice was lower than that of wheat flour. Lowered hardness of the cookies would increase the spread ratio. This would increase the likelihood that the quality of the cookies would be favored by consumers [14,15]. Studies have shown that the low availability of gluten with the multi-millet flour replacement led to a decrease in hardness of the cookies [16]. However, with the colored rice content increasing from 10% to 20%, the hardness of the cookies gradually increased. The trend in hardness was consistent with previous findings that higher content of Chlorella vulgaris increased the hardness from 245.86 N to 330.86 N [17]. They suggested that protein and carbohydrate content of Chlorella vulgaris played a role in water absorption to increase the firmness of cookies. The might be due to the effect of flour with higher dietary fiber on the rate of starch retrogradation during cookies dough preparation.

### 3.3. In Vitro Digestion

**Figure 2:**
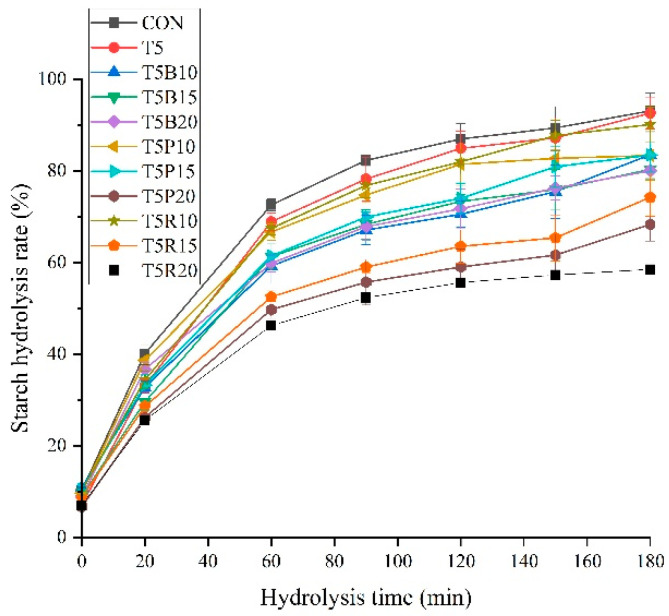
In vitro digestion property of cookies. The test was repeated in triplicate.

The interactions among wheat flour and replacement flour would greatly affect in vitro digestion behavior of starch [18]. The starch hydrolysis rate was depcited in Figure 2. There was no significant (*p* > 0.05) difference among CON, T5, T5P10, and T5R10 samples. However, the starch hydrolysis rate of these groups (CON, T5, T5P10, and T5R10) was significantly (*p* < 0.05) lower than that of other samples from 20 min, and T5R20 sample was the lowest, 58.5% starch hydrolysis rate at 180 min. The combination of *Tricholoma matsutake* mushroom, purple rice, or red rice lowered starch digestion to a certain extent. There was no significant (*p* > 0.05) difference among cookie samples with purple rice flour (T5P10, T5P15, and T5P20 sample) in starch hydrolysis rate. This indicated that 10–20% purple rice flour replacement only slightly affected hydrolysis behavior, whereas, the combination of *Tricholoma matsutake* powder and purple rice would improve the digestive property. Mushroom is an important source of amino acids and bioactive amine. The in vitro intestinal digestion of mushroom could provide significant release of amino acids [19]. Mushroom has the ability to suppress starch crystallization through hydrophobic interactions with starch, and could be used as glycaemic controller of starch-rich foods [20]. Protein from red rice could create a strong protein-starch network to inhibit starch from enzymatic degradation and the hydrolysis rate of T5R20 sample achieved the lowest value [21].

### 3.4. Sensory Attribute

**Figure 3:**
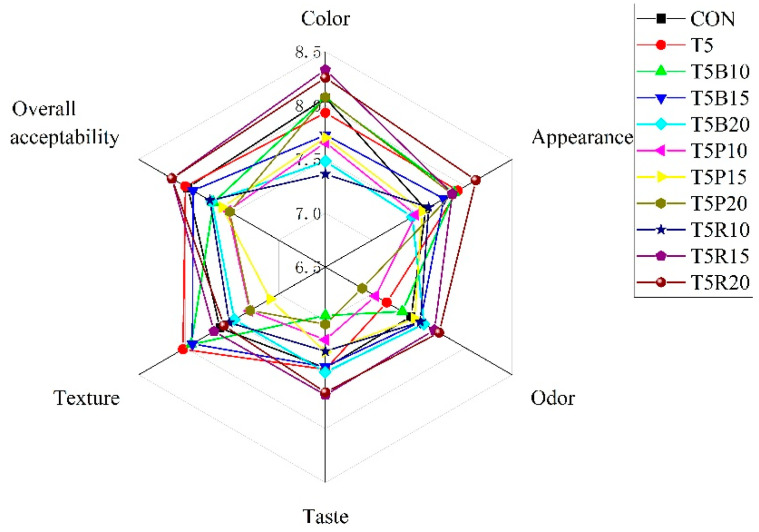
The radar chart of sensory ratings in terms of color, appearance, odor, taste, texture, and overall acceptability. The test was repeated in triplicate.

The sensory ratings of the cookies in terms of color, appearance, odor, taste, texture, and overall acceptability were shown in Figure 3. When compared to the control sample, 5% *Tricholoma matsutake* powder replacement (T5 sample) led to a decrease in color and odor, and an increase in appearance and texture. Similar trend was also found in the cookies with black rice and purple rice substitution. However, with the red rice replacement of wheat flour, the color, taste, texture, and overall acceptability scores of T5R10 sample with 10% red rice was lower than that of the control sample and T5 sample. It was possible that black rice and purple rice were darker than red rice flour, and the T5B20 and T5P20 sample were darker than the T5R20 sample, giving lower acceptance to some reviewers.

With the higher content of red rice replacement, the rating scores gradually increased, and the score of T5R20 sample was the highest. This might be due to the sweet taste from red rice rather than other colored rice. The addition of *Tricholoma matsutake* powder and colored rice flour altered the original texture and appearance of the whole wheat cookies to different degree [12]. Whatever the changes in the sensory rating, the cookie samples were acceptable, especially for the T5R15 and T5R20 sample achieving the highest scores, that most sensory evaluation individuals expressed a preference.

### 3.5. Free Amino Acids

The free amino acids content of eleven cookies samples was listed in Table 3. As could be seen from Table 3, the total content of control sample (304.5 mg/100 g) was the lowest and lower than that of the other cookies samples with mushroom or colored rice replacement. Many researchers reported that mushroom is abundant in free amino acids and provides a strong umami flavor as well as sweetness [2]. The *Tricholoma matsutake* mushroom contains eight essential amino acids, which were threonine (Thr), isoleucine (Ile), valine (Val), leucine (Leu), phenylalanine (Phe), histidine (His), methionine (Met), and lysine (Lys). This also indicated the nutritional potential of *Tricholoma matsutake* mushroom as a source of essential amino acids [22].

The total content of T5 sample with 5% *Tricholoma matsutake* powder replacement was 563.3 mg/100 g, and that of T5B20, T5P20, and T5R20 cookie samples with 20% colored rice replacement was 559.3, 580.7, and 409.9 mg/100 g, respectively. For cookie samples with 5% *Tricholoma matsutake* powder replacement, the black rice or purple rice substitution did not significantly (*p* > 0.05) affect the free amino acid content, whereas the red rice substitution significantly (*p* < 0.05) lowered the free amino acid content. This might be due to the fact that colored rice did not mainly provide amino aroma for cookies. However, the tyrosine content of red rice was significantly (*p* < 0.05) lower than that of black rice or purple rice. During cookie baking, the free amino acids would involve in the Maillard reaction, which lead to the decrease in amino acid content. Meanwhile, the protein breakdown might produce free amino acids [23].

As could be known from Table 3, the identified free amino acids were divided into four taste types (umami, sweet, bitter, and tasteless) [24]. The umami content of cookie samples with 5% *Tricholoma matsutake* was significantly (*p* < 0.05) higher than that of control group. However, there was no significantly (*p* > 0.05) difference in the umami content between the cookies with *Tricholoma matsutake* powder or colored rice (For example, 39.4 mg/100 g for T5 sample, and 38.1 mg/100 g for T5B20 sample). Asp showed significantly (*p* < 0.05) lower content than Glu. Glu and Asp mainly contributed to umami and mushroom taste, which were the characteristic and pleasant flavor of mushrooms [25]. Glu is an acidic amino acid with two carboxyl groups and could prevent from interacting with reducing carbohydrate [26]. The Glu amino acid mainly contributed to umami taste for cookie samples with *Tricholoma matsutake* mushroom, since its content was high above the thresholds, 17.8 mg/100 g for Glu [27].

Ser, Ala, Thr, and Gly are included in the sweet taste. The sweet content of control sample (16.0 mg/100 g) was the lowest and significantly (*p* < 0.05) lower than that of the other cookie samples with mushroom or colored rice bi 49.7–69.5 mg/100 g). The free amino acids of mushroom provide a strong sweetness flavor. The cookies with red rice significantly (*p* < 0.05) increased the free amino acid content of sweet taste. This indicated that red rice could further improve the sweetness of cookies.

Cys and Met are sulfur-containing amino acids. Cys significantly (*p* < 0.05) affected the content of tasteless amino acids, whereas Met was in a low level (0.78–2.20 mg/100 g) for all samples. Cys could generate sulfur-containing flavor compounds, such as 2-methyl-3-thiophenethiol, 3-mercapto-2-butanone, and 2-butanone, through Maillard reaction [28].

### 3.6. Aroma Analysis of Cookies

**Figure 4:**
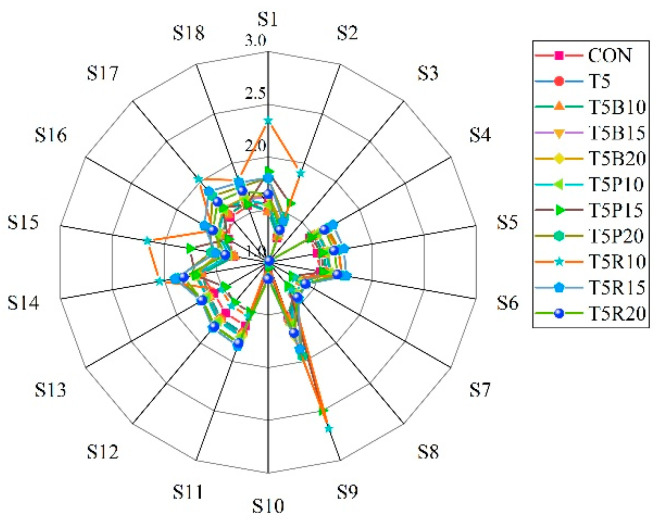
The radar chart of cookies odor evaluation by eighteen e-nose sensors. The test was repeated in triplicate.

Aroma is very important for consumer evaluation and acceptance of bread. The aroma evaluation by the radar chart was displayed in Figure 4. E-nose technology can objectively and rapidly recognize the overall volatile profile of food products [29]. The system used in this study contains 18 metal oxide sensors as listed in Table 4, and the response value of each sensor is representative of different VOCs compound, manifesting the difference between volatile compounds of the cookies. An odor fingerprint was established based on the electronic nose data for 11 cookie samples.

As could be seen from Figure 4, the E-nose sensors including S1, S4, S5, S6, S9, S11, S12, S13, S14, and S17 exhibited relatively higher response value to the volatile compounds, and the highest response was observed in S9 sensor. *Tricholoma matsutake* mushroom contained elevated level of some volatile compounds, such as hexanal, heptanal, 2(5H)-furanone, acetophenone, nonanal, and benzeneacetaldehyde [6]. A distinct change in volatile odor with *Tricholoma matsutake* mushroom or colored rice addition was beneficial to production of volatile substances corresponding to these sensors. The E-nose sensors including S1, S2, S4, S5, S6, S9, S15, S16, S17, and S18 sensors of T5R20 group were relatively higher than those for T5B20 group and T5P20 group. Red rice replacement for wheat flour improved the aroma of cookies better than black rice and purple rice. The decomposition of ester and the reduction in aldehyde usually produce alcohol. Furthermore, the metabolism of abundant amino acids in *Tricholoma matsutake* mushroom would produce flavor precursor, which might affect the flavor of bakery food [30,31].

**Figure 5:**
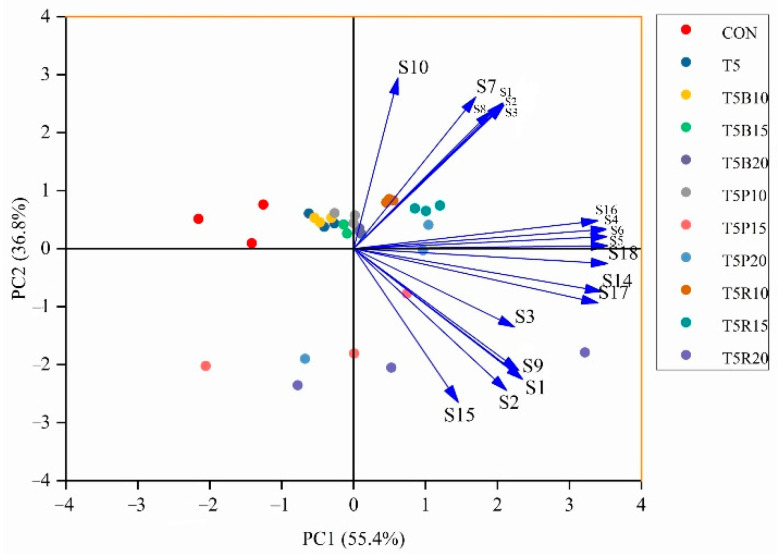
The PCA images for cookie aroma. Arrows represent the loadings of different variables, indicating their contribution to the principal components PC1 and PC2. PC1 explains 55.4% of the variance, while PC2 explains 36.8%.

The Principal Component Analysis (PCA) for cookie aroma was shown in Figure 5. The dimension reduction in E-nose data could be achieved by PCA method, and the correlation among multiple variables was also could be identified [32]. Similar aroma characteristic was approach or overlapping, however, apparent difference between samples was of larger distance [33]. The PCA images in Figure 4. showed that PC1 and PC2 were accounted for 55.4% and 36.8%, respectively, with 92.2% cumulative contribution at the 95% confidence interval. The cumulative contribution higher than 85% indicated the response value of the volatile compounds originated from the E-nose sensors was reliable.

The PCA results showed that 18 sensors were divided into six groups. The first group was S10; the second group was S7, S8, and S12; the fourth group was consisted of S14 and S17; the fifth group was S3; the sixth group was S2, S9, and S15; and the rest sensors were of the third group. This manifested that PCA effectively captured most of the volatile odor characteristics of the cookie samples and the result of E-nose was adequate to identify the similarities among samples [34]. The quality of cookie was closely connected with its volatile odor characteristics and consistent with the result in sensory evaluation.

### 3.7. Pearson Correlation Analysis

**Figure 6:**
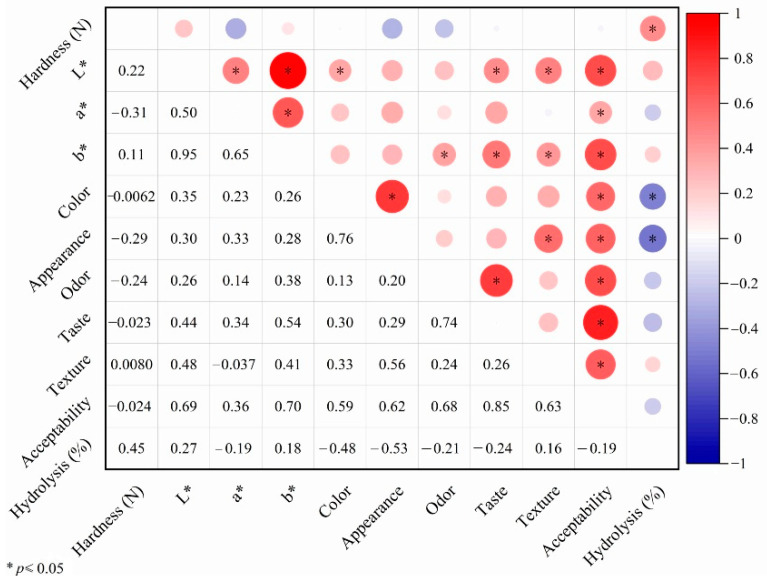
The Pearson correlation among hardness, color, sensory score, and starch hydrolysis rate. Correlation matrix showing the relationships between different attributes. The size of each circle represents the strength of the correlation, with larger circles indicating stronger correlations. Color indicates the direction of the correlation, with red representing positive correlations and blue representing negative correlations. Asterisks denote significance levels: *p* ≤ 0.05.

The Pearson correlation among hardness, color, sensory score, and starch hydrolysis rate was shown in Figure 6. L* value was significantly (*p* < 0.05, r = 0.69) positively correlated with overall acceptance. The b* value and a* value (*p* < 0.05, r = 0.65), L* value (*p* < 0.05, r = 0.95), and overall acceptance (*p* < 0.05, r = 0.70) were also significantly positively correlated. The cookie color was significantly positively correlated with appearance (*p* < 0.05, r = 0.76) and overall acceptance (*p* < 0.05, r = 0.59) of sensory attributes. The lightness of *Tricholoma matsutake* mushroom and colored rice flour affected the brown color of cookies and the acceptance of consumers. The odor score was significantly positively correlated with taste (*p* < 0.05, r = 0.74) and overall acceptance (*p* < 0.05, r = 0.68) of sensory attributes. *Tricholoma matsutake* mushroom is abundant in free amino acids, which provides popular odor for consumers. This indicated that *Tricholoma matsutake* mushroom substitution would offer a preferred flavor to cookies. The overall acceptance was positively related to several attributes, such as L* value, b* value, color score, appearance, odor, taste, and texture of cookies, which would significantly affect the consumer’s purchase intentions. This proved the alternative for *Tricholoma matsutake* mushroom and colored rice flour to improve quality of the cookies.

## 4. Conclusions

The quality feature of cookies with *Tricholoma matsutake* mushroom powder and colored rice flour substitution was evaluated herein. A 5% replacement amount of Tricholoma matsutake mushroom powder combined with a 20% replacement amount of colored rice flour gave the cookies a brownish color and moderate hardness that falls within consumers’ acceptance range. As for the cookies with colored rice substitution, the peak temperature only slightly shifted. The wheat flour substitution with higher content of dietary fiber or straight-chain starch only slightly affected the crystallinity of the starch granules. Sensory evaluation for the T5R15 and T5R20 sample achieved the highest scores, compared to the other samples. The mushroom or colored rice replacement increased the free amino acid content of cookies and endowed a special and popular smell and taste for cookies. According to the current results, the combination of wheat flour with the *Tricholoma matsutake* mushroom powder and colored rice flour may have the potential for the development of functional cookies. The developed cookies may be an alternative product for consumers with reduced caloric and glycemic demands.

## Figures and Tables

**Table 1:** The recipe of cookies.

No.	1	2	3	4	5	6	7	8	9	10	11
Cookie (g)	CON	T5	T5B10	T5B15	T5B20	T5P10	T5P15	T5P20	T5R10	T5R15	T5R20
*Tricholoma matsutake* (g)	0	7.5	7.5	7.5	7.5	7.5	7.5	7.5	7.5	7.5	7.5
Colored rice (g)	0	0	15	22.5	30	15	22.5	30	15	22.5	30
Low-gluten flour (g)	130	122.5	107.5	100	92.5	107.5	100	92.5	107.5	100	92.5
High-gluten flour (g)	20	20	20	20	20	20	20	20	20	20	20
Butter (g)	120	120	120	120	120	120	120	120	120	120	120
Water (g)	10	10	10	10	10	10	10	10	10	10	10
Milk powder (g)	10	10	10	10	10	10	10	10	10	10	10
Sugar (g)	50	50	50	50	50	50	50	50	50	50	50
Salt (g)	2	2	2	2	2	2	2	2	2	2	2
Egg (g)	25	25	25	25	25	25	25	25	25	25	25

CON denotes control cookie without substitution; T5 denotes cookie with 5% *Tricholoma matsutake* powder substitution; T5B10, T5B15, and T5B20 denote cookies with 5% *Tricholoma matsutake* powder and 10, 15, and 20% black rice substitution; T5P10, T5P15, and T5P20 denote cookies with 5% *Tricholoma matsutake* powder and 10, 15, and 20% purple rice substitution; T5R10, T5R15, and T5R20 denote cookies with 5% *Tricholoma matsutake* powder and 10, 15, and 20% red rice substitution, respectively.

**Table 2:** The physical properties of cookies.

No.	1	2	3	4	5	6	7	8	9	10	11
Cookie	CON	T5	T5B10	T5B15	T5B20	T5P10	T5P15	T5P20	T5R10	T5R15	T5R20
	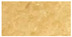	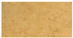	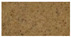	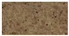	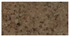	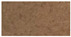	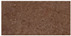	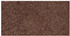	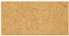	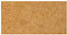	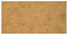
L*	74.13 ± 0.89 ^e^	68.75 ± 1.23 ^d^	55.79 ± 0.58 ^b^	55.91 ± 1.37 ^b^	48.27 ± 1.87 ^a^	54.89 ± 1.28 ^b^	47.92 ± 2.37 ^a^	48.99 ± 1.82 ^a^	65.29 ± 1.09 ^c^	65.77 ± 1.01 ^c^	65.17 ± 0. 70 ^c^
a*	4.75 ± 0.11 ^abc^	7.01 ± 0.37 ^de^	3.95 ± 0.24 ^ab^	3.60 ± 0.86 ^a^	3.47 ± 0.90 ^a^	6.05 ± 0.69 ^cd^	5.07 ± 0.42 ^bc^	5.36 ± 0.42 ^bc^	7.92 ± 0.61 ^e^	8.24 ± 0.63 ^e^	7.79 ± 0.25 ^e^
b*	35.08 ± 0.84 ^g^	33.96 ± 0.82 ^ef^	21.55 ± 0.76 ^cd^	20.23 ± 0.38 ^bc^	19.65 ± 0.82 ^b^	22.31 ± 0.60 ^d^	16.44 ± 0.53 ^a^	15.57 ± 0.91 ^a^	34.75 ± 1.22 ^ef^	33.47 ± 1.06 ^e^	33.37 ± 0.10 ^e^
Hardness (g)	1602 ± 147 ^d^	1194 ± 74 ^c^	590 ± 20 ^a^	782 ± 13 ^a^	1278 ± 31 ^c^	678 ± 22 ^a^	762 ± 18 ^a^	971 ± 12 ^b^	589 ± 35 ^a^	619 ± 31 ^a^	664 ± 12 ^a^

CON denotes control cookie without substitution; T5 denotes cookie with 5% *Tricholoma matsutake* powder substitution; T5B10, T5B15, and T5B20 denote cookies with 5% *Tricholoma matsutake* powder and 10, 15, and 20% black rice substitution; T5P10, T5P15, and T5P20 denote cookies with 5% *Tricholoma matsutake* powder and 10, 15, and 20% purple rice substitution; T5R10, T5R15, and T5R20 denote cookies with 5% *Tricholoma matsutake* powder and 10, 15, and 20% red rice substitution, respectively. Means within rows with different letters are significantly different (*t* test, *p* < 0.05).

**Table 3:** The free amino acid content (mg/100 g dry weight) of eleven cookie samples. The test was repeated in triplicate.

No.	Compounds	Abb.	1	2	3	4	5	6	7	8	9	10	11
			CON	T5	T5B10	T5B15	T5B20	T5P10	T5P15	T5P20	T5R10	T5R15	T5R20
Umami	Glutamic acid	Glu	9.98 ± 0.14 ^a^	31.4 ± 0.34 ^b^	33.4 ± 0.21 ^b^	29.8 ± 0.61 ^b^	30.7 ± 0.18 ^b^	30.5 ± 0.52 ^b^	33.2 ± 0.10 ^b^	35.3 ± 1.08 ^b^	29.5 ± 0.21 ^b^	32.3 ± 0.13 ^b^	31.7 ± 1.59 ^b^
	Aspartic acid	Asp	5.32 ± 0.58 ^a^	7.98 ± 0.51 ^c^	7.81 ± 0.36 ^c^	7.45 ± 1.19 ^bc^	7.39 ± 0.67 ^bc^	7.38 ± 0.35 ^bc^	8.03 ± 0.51 ^c^	8.33 ± 0.21 ^d^	6.87 ± 0.78 ^b^	7.52 ± 0.61 ^bc^	7.57 ± 0.10 ^bc^
Subtotal			15.3 ± 0.64 ^a^	39.38 ± 0.85 ^b^	41.21 ± 0.57 ^bc^	37.25 ± 1.80 ^b^	38.09 ± 0.81 ^b^	37.88 ± 0.34 ^b^	41.23 ± 0.61 ^bc^	43.63 ± 1.20 ^c^	36.37 ± 0.80 ^b^	39.82 ± 0.71 ^b^	39.27 ± 1.54 ^b^
Sweet	Serine	Ser	1.04 ± 0.02 ^a^	11.9 ± 0.06 ^b^	11.9 ± 0.01 ^b^	10.9 ± 0.02 ^b^	11.2 ± 0.00 ^b^	10.7 ± 0.07 ^b^	11.5 ± 0.00 ^b^	12.0 ± 0.11 ^b^	10.3 ± 0.06 ^b^	11.4 ± 0.02 ^b^	11.3 ± 0.03 ^b^
	Alanine	Ala	7.67 ± 0.11 ^a^	8.45 ± 0.02 ^b^	8.27 ± 0.08 ^b^	7.67 ± 0.04 ^a^	7.35 ± 0.13 ^a^	7.69 ± 0.11 ^a^	19.8 ± 0.21 ^c^	8.89 ± 0.15 ^b^	18.7 ± 0.10 ^c^	20.8 ± 0.08 ^c^	19.4 ± 0.07 ^c^
	Threonine	Thr	6.43 ± 0.08 ^a^	34.4 ± 0.02 ^cd^	37.0 ± 0.03 ^d^	32.1 ± 0.07 ^c^	31.0 ± 0.16 ^c^	31.4 ± 0.02 ^c^	32.1 ± 0.08 ^c^	37.6 ± 0.01 ^d^	28.7 ± 0.10 ^b^	27.5 ± 0.05 ^b^	29.7 ± 0.00 ^b^
	Glycine	Gly	0.89 ± 0.07 ^a^	0.15 ± 0.16 ^a^	0.35 ± 0.01 ^a^	0.36 ± 0.00 ^a^	0.18 ± 0.02 ^a^	0.33 ± 0.01 ^a^	6.09 ± 0.10 ^b^	0.22 ± 0.08 ^a^	6.11 ± 0.02 ^b^	6.86 ± 0.12 ^b^	6.27 ± 0.10 ^b^
Subtotal			16.03 ± 0.30 ^a^	54.9 ± 0.25 ^bc^	57.52 ± 0.14 ^bc^	51.03 ± 0.14 ^b^	49.73 ± 0.23 ^b^	50.12 ± 0.20 ^b^	69.49 ± 0.35 ^d^	58.71 ± 0.35 ^bc^	63.81 ± 0.27 ^c^	66.56 ± 0.25 ^cd^	66.67 ± 0.20 ^cd^
Bitter	Isoleucine	Ile	2.34 ± 0.09 ^a^	4.95 ± 0.06 ^b^	5.04 ± 0.01 ^b^	4.68 ± 0.06 ^b^	4.63 ± 0.00 ^b^	4.50 ± 0.06 ^b^	4.81 ± 0.01 ^b^	5.34 ± 0.04 ^b^	4.74 ± 0.02 ^b^	5.78 ± 0.02 ^b^	5.35 ± 0.32 ^b^
	Valine	Val	4.43 ± 0.20 ^a^	12.2 ± 0.36 ^b^	12.3 ± 0.02 ^b^	10.9 ± 0.24 ^b^	10.9 ± 0.51 ^b^	11.0 ± 0.00 ^b^	11.5 ± 0.21 ^b^	12.4 ± 0.06 ^b^	11.3 ± 0.01 ^b^	13.0 ± 0.05 ^bc^	12.1 ± 0.01 ^b^
	Leucine	Leu	4.94 ± 1.09 ^a^	8.67 ± 0.08 ^b^	9.17 ± 1.06 ^b^	8.83 ± 0.22 ^b^	9.18 ± 0.21 ^b^	9.32 ± 0.02 ^b^	12.6 ± 0.01 ^c^	9.67 ± 0.03 ^b^	12.0 ± 0.00 ^c^	13.5 ± 0.02 ^c^	13.4 ± 0.03 ^c^
	Arginine	Arg	1.92 ± 0.36 ^a^	2.24 ± 0.24 ^b^	3.17 ± 0.02 ^b^	2.47 ± 0.24 ^b^	2.66 ± 0.05 ^b^	2.66 ± 0.03 ^b^	2.45 ± 0.00 ^b^	2.93 ± 0.02 ^b^	2.91 ± 0.16 ^b^	2.74 ± 0.08 ^b^	2.61 ± 0.02 ^b^
	Phenylalanine	Phe	19.6 ± 0.12 ^b^	21.4 ± 0.22 ^b^	20.7 ± 0.24 ^b^	21.6 ± 0.13 ^b^	22.2 ± 0.01 ^c^	18.1 ± 0.16 ^a^	17.4 ± 0.06 ^a^	18.8 ± 0.16 ^a^	17.1 ± 0.01 ^a^	18.7 ± 0.00 ^a^	16.1 ± 0.19 ^a^
	Histidine	His	39.9 ± 0.14 ^b^	70.8 ± 0.13 ^c^	72.3 ± 0.99 ^cd^	68.0 ± 0.23 ^c^	79.2 ± 0.04 ^d^	63.9 ± 0.02 ^bc^	69.1 ± 1.34 ^c^	71.1 ± 0.02 ^c^	8.01 ± 0.04 ^a^	9.31 ± 0.01 ^a^	8.69 ± 0.28 ^a^
	Methionine	Met	2.20 ± 0.07 ^a^	1.22 ± 0.16 ^a^	1.08 ± 0.30 ^a^	0.78 ± 0.39 ^a^	0.88 ± 0.02 ^a^	1.07 ± 0.03 ^a^	1.06 ± 0.07 ^a^	1.29 ± 0.31 ^a^	1.22 ± 0.25 ^a^	1.10 ± 0.21 ^a^	1.41 ± 0.03 ^a^
Subtotal			75.33 ± 0.52 ^b^	121.48 ± 0.41 ^de^	123.76 ± 0.89 ^de^	117.26 ± 0.51 ^d^	129.65 ± 0.14 ^e^	110.55 ± 0.23 ^c^	118.92 ± 0.14 ^d^	121.53 ± 0.41 ^de^	57.28 ± 0.28 ^a^	64.13 ± 0.64 ^ab^	59.66 ± 0.99 ^a^
Tasteless	Tyrosine	Tyr	50.2 ± 0.09 ^b^	53.1 ± 0.36 ^bc^	53.2 ± 0.10 ^bc^	52.1 ± 0.24 ^b^	52.6 ± 0.07 ^b^	54.0 ± 0.19 ^c^	51.7 ± 0.32 ^b^	54.4 ± 0.03 ^c^	12.4 ± 0.14 ^a^	12.7 ± 0.19 ^a^	12.2 ± 0.02 ^a^
	Lysine	Lys	16.6 ± 2.11 ^a^	20.7 ± 1.80 ^c^	21.7 ± 0.36 ^c^	20.6 ± 0.03 ^c^	19.5 ± 0.83 ^bc^	20.0 ± 0.26 ^c^	18.4 ± 0.11 ^a^	20.2 ± 0.37 ^c^	18.6 ± 0.03 ^a^	21.6 ± 0.32 ^c^	20.0 ± 0.26 ^c^
	Cysteine	Cys	24.4 ± 1.09 ^a^	58.0 ± 0.68 ^d^	58.5 ± 1.14 ^d^	49.3 ± 0.30 ^bc^	52.3 ± 0.11 ^c^	48.2 ± 0.22 ^bc^	52.5 ± 0.03 ^c^	58.4 ± 0.18 ^d^	46.2 ± 0.32 ^b^	46.1 ± 0.01 ^b^	46.5 ± 0.03 ^b^
Subtotal			91.2 ± 2.28 ^b^	131.8 ± 2.26 ^d^	133.4 ± 1.91 ^d^	122.0 ± 0.63 ^c^	124.4 ± 0.90 ^c^	122.2 ± 0.64 ^c^	122.6 ± 0.17 ^c^	133.0 ± 0.94 ^d^	77.2 ± 0.69 ^a^	80.4 ± 0.57 ^ab^	78.7 ± 0.32 ^a^
Total			304.52 ± 1.92 ^a^	563.32 ± 1.95 ^de^	578.38 ± 0.16 ^e^	533.08 ± 0.19 ^de^	559.34 ± 0.63 ^d^	519.3 ± 0.82 ^d^	581.88 ± 0.36 ^e^	580.74 ± 1.25 ^e^	392.12 ± 1.96 ^c^	421.42 ± 0.86 ^bc^	409.9 ± 0.36 ^bc^

CON denotes control cookie without substitution; T5 denotes cookie with 5% *Tricholoma matsutake* powder substitution; T5B10, T5B15, and T5B20 denote cookies with 5% *Tricholoma matsutake* powder and 10, 15, and 20% black rice substitution; T5P10, T5P15, and T5P20 denote cookies with 5% *Tricholoma matsutake* powder and 10, 15, and 20% purple rice substitution; T5R10, T5R15, and T5R20 denote cookies with 5% *Tricholoma matsutake* powder and 10, 15, and 20% red rice substitution, respectively. Means within rows with different letters are significantly different (*t* test, *p* < 0.05).

**Table 4:** Sensor and performance of E-nose.

Sensor	Object Substances for Sensing
S1	Propane, smoke, etc.
S2	Alcohol, smoke, isobutane, formaldehyde, etc.
S3	Hydrogen
S4	Sulfides
S5	Ammonia, amines, etc.
S6	Toluene, acetone, ethanol, hydrogen, etc.
S7	Short-chain alkane combustible gases, etc.
S8	Liquefied gas
S9	Toluene, formaldehyde, benzene, alcohol, acetone, etc.
S10	Hydrogen-containing gases, etc.
S11	Alkanes, carbon monoxide, etc.
S12	Liquefied gas, methane, etc.
S13	Short-chain alkanes, etc.
S14	Methane, gas, smoke, etc.
S15	Ammonia, amines, etc.
S16	Hydrogen sulfide
S17	Hydrogen-containing substances
S18	Aromatic hydrocarbons, aliphatic hydrocarbons, alicyclic hydrocarbons, halogenated hydrocarbons, ethers, esters, diol derivatives, acetonitrile, pyridine, phenol, etc.

## Data Availability

The original contributions presented in the study are included in the article; further inquiries can be directed to the corresponding author.
